# Comparative effects of lipid lowering, hypoglycemic, antihypertensive and antiplatelet medications on carotid artery intima-media thickness progression: a network meta-analysis

**DOI:** 10.1186/s12933-019-0817-1

**Published:** 2019-01-30

**Authors:** Rongzhong Huang, Kerry Mills, Julio Romero, Yan Li, Zicheng Hu, Yu Cao, Hua Huang, Yu Xu, Lihong Jiang

**Affiliations:** 1 Department of Cardiothoracic Surgery, The First People’s Hospital of Yunnan Province, Kunming, Yunnan China; 2 Health Research Institute, University of Canberra, Kirinari St, Bruce, ACT 2617 Australia; 3 Department of Software Engineering and Artificial Intelligence, University of Canberra, Canberra, Australia; 4 Department of Mathematics and Statistics, University of Canberra, Canberra, Australia; 5 Statistical Laboratory, Chuangxu Institute of Life Science, Chongqing, China; 6 Department of Geriatrics, The First People’s Hospital of Yunnan Province, No. 157 Jinbi Road, Kunming, 650000 Yunnan China; 7 Department of Neurology, Institute of Surgery Research, Daping Hospital, Third Military Medical University, Chongqing, China; 8 Department of Neurology, The Second Affiliated Hospital of Chongqing Medical University, Chongqing, China

**Keywords:** Atherosclerosis, Intima-media thickness, Metabolic disorders, Cardiovascular, Diabetes

## Abstract

**Background:**

Carotid artery intima-media thickness (cIMT) progression is a surrogate marker of atherosclerosis with a high predictive value for future CVD risk. This study evaluates the comparative efficacies of lipid lowering, hypoglycemic, antihypertensive and antiplatelet medications on cIMT progression.

**Methods:**

We conducted a network meta-analysis (NMA) to evaluate the relative efficacies of several drug classes in modifying cIMT progression. After a literature search in several electronic databases, studies were selected by following predetermined eligibility criteria. An inverse variance-heterogeneity model was used for NMA. Sensitivity analyses were performed to check the reliability of the overall NMA, and transitivity analyses were performed to examine the effects of modifiers on the NMA outcomes.

**Results:**

Data were taken from 47 studies (15,721 patients; age: 60.2 years [95% confidence interval (CI) 58.8, 61.6]; BMI: 27.2 kg/m^2^ [95% CI 26.4, 28.0]; and gender: 58.3% males [95% CI 48.3, 68.3]). Treatment duration was 25.8 months [95% CI 22.9, 28.7]. Of the 13 drug classes in the network, treatment with phosphodiesterase III inhibitors was the most effective in retarding annual mean cIMT against network placebo (weighted mean difference (WMD) − 0.059 mm [95% CI − 0.099, − 0.020) followed by the calcium channel blockers (WMD − 0.055 mm [95% CI − 0.099, 0.001]) and platelet adenosine diphosphate inhibitors (WMD − 0.033 mm [95% CI − 0.058, 0.008]). These 3 drug classes also attained the same positions when the NMA was conducted by using first-year changes in mean cIMT. In transitivity analyses, longer treatment duration, higher body mass index (BMI), and a higher baseline cIMT were found to be independently associated with a lesser reduction in annual mean cIMT. However, in a multivariate analysis with these 3 modifiers, none of these factors was significantly associated with annual change in mean cIMT. In the placebo group, age was inversely associated with annual change in mean cIMT independently.

**Conclusion:**

Phosphodiesterase III inhibitors and calcium channel blockers are found more effective than other drug classes in retarding cIMT progression. Age, BMI, and baseline cIMT may have some impact on these outcomes.

**Electronic supplementary material:**

The online version of this article (10.1186/s12933-019-0817-1) contains supplementary material, which is available to authorized users.

## Introduction

Metabolic diseases constitute a major global public health challenge. An aggregation of conditions like visceral obesity, dyslipidemia, hyperglycemia, hypertension, and insulin resistance also makes an individual vulnerable to type 2 diabetes and cardiovascular disease (CVD) [1, 2]. One of the most important associations of metabolic diseases is atherosclerosis, which is difficult to detect in younger healthy populations because of its gradual progression perhaps continuing throughout life depending on the presence of one or more risk factors [3]. Atherosclerosis risk factors include male gender, old age, smoking, hypertension, dyslipidemia (hypertriglyceridemia, higher low-density lipoproteins (LDL)-cholesterol and lower high-density lipoprotein (HDL)-cholesterol), hyperinsulinemia, family history, obesity, and diabetes mellitus [4].

A surrogate marker of atherosclerosis is intima-media thickness (IMT), which is the combined thickness of the tunica intima and media of a circulatory vessel detectable non-invasively with ultrasonographic techniques [5]. It is a valuable indicator of CVD risk, significantly correlated with current and future CVD when its absolute value and progression rates are determined [6]. Based on data from many studies, carotid artery IMT (cIMT) progression is considered an indicator of atherosclerosis with a high predictive value for future CVD and related mortality [7].

Several drugs are used to treat diabetes and other metabolic diseases, and evaluation of their efficacies is an active area of clinical research. Several trials have also reported the effect of various drugs on cIMT dynamics. We have undertaken a systematic review of relevant trials which evaluated the efficacy of one or more drug/s and reported on cIMT changes. Data acquired from these studies were used to conduct network meta-analysis (NMA) for evaluating the relative efficacies of different drug classes in changing mean cIMT.

## Method

### Eligibility criteria

A study was included if it (a) evaluated the efficacy of a drug/s used for the management of metabolic diseases (antihypertensive, antiplatelet, hypoglycemic, lipid lowering) in changing mean cIMT; (b) recruited patients with diabetes mellitus, impaired glucose tolerance, CVD, or metabolic syndrome and treated patients for at least 6 months; (c) reported annual changes in mean cIMT or baseline, yearly and end of study mean cIMT values; (d) had a placebo or comparative drug control group to compare the outcomes; and (e) investigated one or more of the following classes of drugs—alpha-glucosidase inhibitors, angiotensin I converting enzyme (ACE) inhibitor, angiotensin II receptor blockers, beta blockers, cyclooxygenase inhibitors, calcium channel blockers, dipeptidyl peptidase-4 (DPP4) inhibitors, glucagon-like peptide 1 (GLP-1) analogues, hydroxy-methylglutaryl coenzyme A (HMG CoA) reductase inhibitors, incretin-based therapies, insulin secretagogues, peroxisome proliferator-activated receptor (PPAR) alpha/gamma agonists, phosphodiesterase III inhibitors, sodium-chloride cotransporter inhibitors, or sodium-glucose cotransporter-2 (SGLT2) inhibitors.

A study was excluded if it (a) compared a nutrient rather than a drug against a placebo or a comparative drug, (b) used a combination of drugs in one or both arms, (c) was a single arm trial, or (d) reported an endpoint other than mean cIMT (e.g., maximum IMT, etc.).

### Literature search

The literature search was conducted out in electronic databases (Embase, Google Scholar, Ovid SP, and PubMed). Important Medical Subject Headings and key terms were used as combination phrases. Primary combination phrases included: carotid intima-media thickness—atherosclerosis—drug therapy. In a secondary search, the primary combination was used with several other keywords. The full search strategy is given in Additional file 1: Appendix S1. Bibliographies of important research and review articles, as well as database corroborations were also screened.

### Data and analyses

Required cIMT measurement data along with important demographic and clinical information about the patients and study characteristics were extracted from the research articles and synthesized in datasheets for use in the NMA. The endpoints for NMAs were (a) annual change in mean cIMT and (b) first-year change in mean cIMT. NMAs were performed with MetaXL software (version 5.3; EpiGear International Pty Ltd) using continuous data under the inverse variance-heterogeneity (IVhet) model. Direct and repeated adjusted indirect comparisons were based on Generalized Pairwise Modeling (GPM) framework under the assumption that common control nodes are sufficiently similar [8, 9]. We used weighted mean differences as an effect estimator. IVhet is a distributional assumption-free model that tends to yield model-based estimator variance closer to the observed variance by re-scaling overdispersion. For this, IVhet adapts a quasi-likelihood approach in which individual comparisons are performed under a fixed effect assumption (inverse variance weighting) but the variance of the overall effect estimate is inflated to account for the heterogeneity [9].

As sensitivity analyses, NMAs were performed by using placebo-controlled studies (no comparator-controlled arms) under the IVhet model with MetaXL software and a random-effects conventional meta-analysis of mean differences between active drugs and their placebos in changing annual mean cIMT was performed with RevMan software (version 5.3; Cochrane Collaboration).

To assess the quality of the included studies, the Cochrane Collaboration’s Tool for the Quality Assessment of Randomized Controlled Trials was used. Assessment of publication bias was performed with Begg’s test. Transitivity analyses were performed to evaluate the impact of modifiers on NMA outcomes (annual change in mean cIMT) [10]. For this purpose, the effect sizes of all treatment and placebo arms were pooled separately and were subjected to metaregression with Stata software (Stata Corporation; Texas, USA) to evaluate the strength of associations between outcomes and modifiers.

## Results

### Data characteristics

**Fig. 1:**
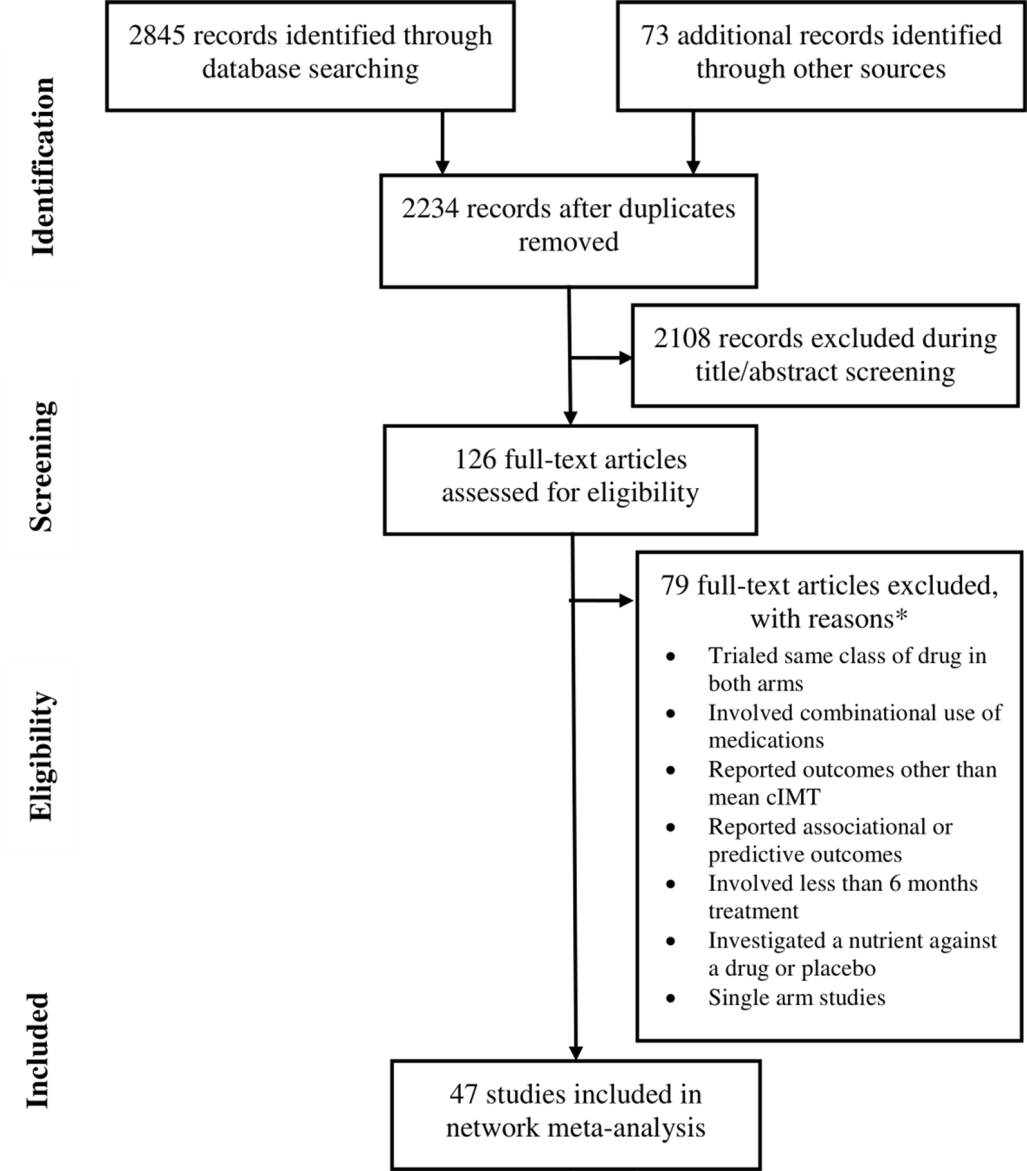
A flowchart of the study screening and selection process

Forty-seven studies [11–57] were selected for inclusion in the NMA (Fig. 1). Available datasets (trial arms) were 53. Important characteristics of the included studies are presented in Additional file 1: Table S1. In these studies, 10,254 patients were treated with a therapeutic drug and 5467 patients were treated with placebo. The age and body mass index (BMI) of these patients were 60.2 years [95% confidence interval (CI): 58.8, 61.6] and 27.2 kg/m^2^ [95% CI 26.4, 28.0], respectively. In this population, the percentage of males was 58.3% [95% CI 48.3, 68.3] and the percentage of current smokers was 29.2% [95% CI 22.4, 35.9]. This population consisted of patients with type 2 diabetes mellitus (48.6%), dyslipidemia (18.7%), coronary syndromes (12.8%), hypertension (12.05%), and impaired glucose tolerance (7.8%).

### NMA outcomes

**Fig. 2:**
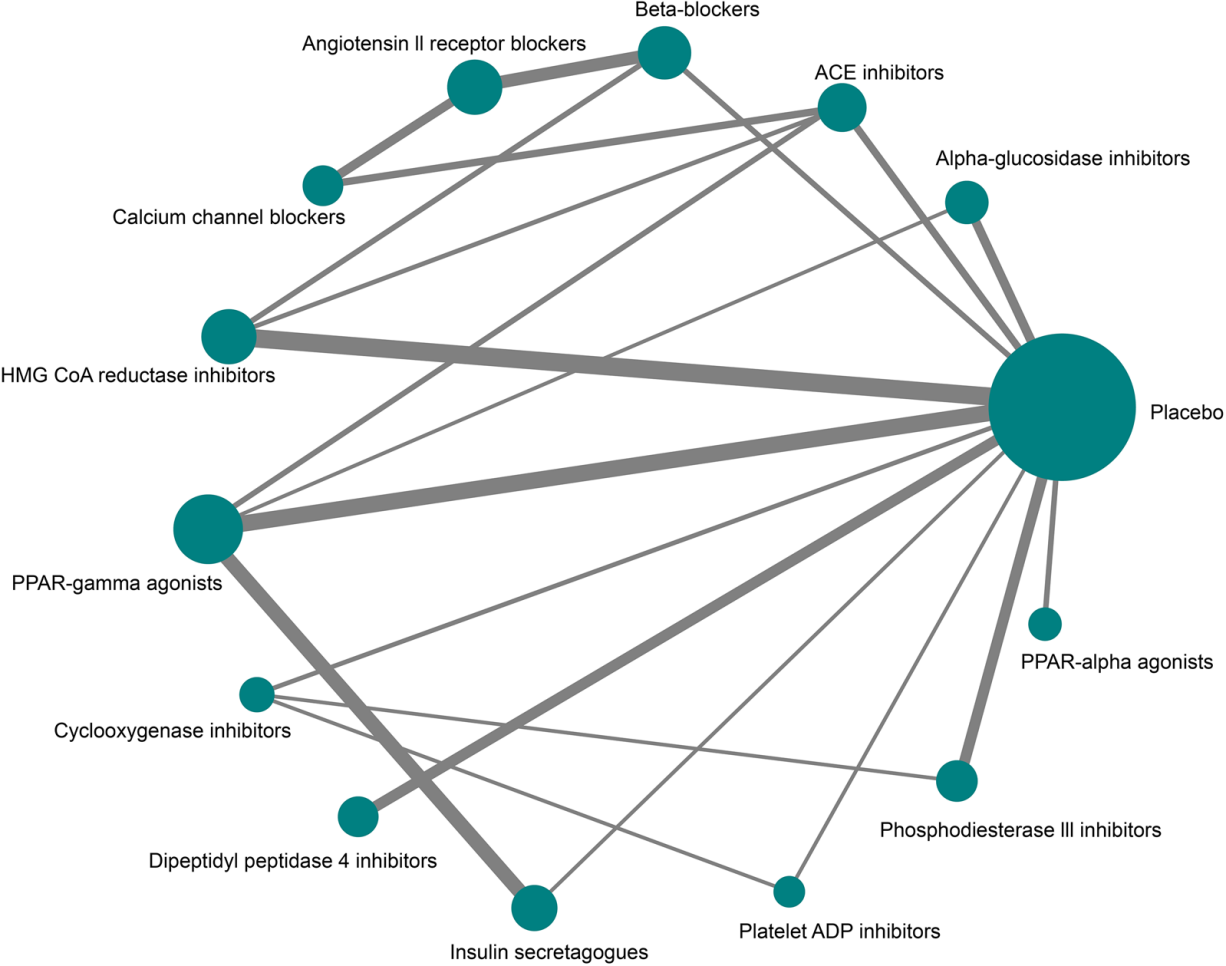
Network diagram

**Fig. 3:**
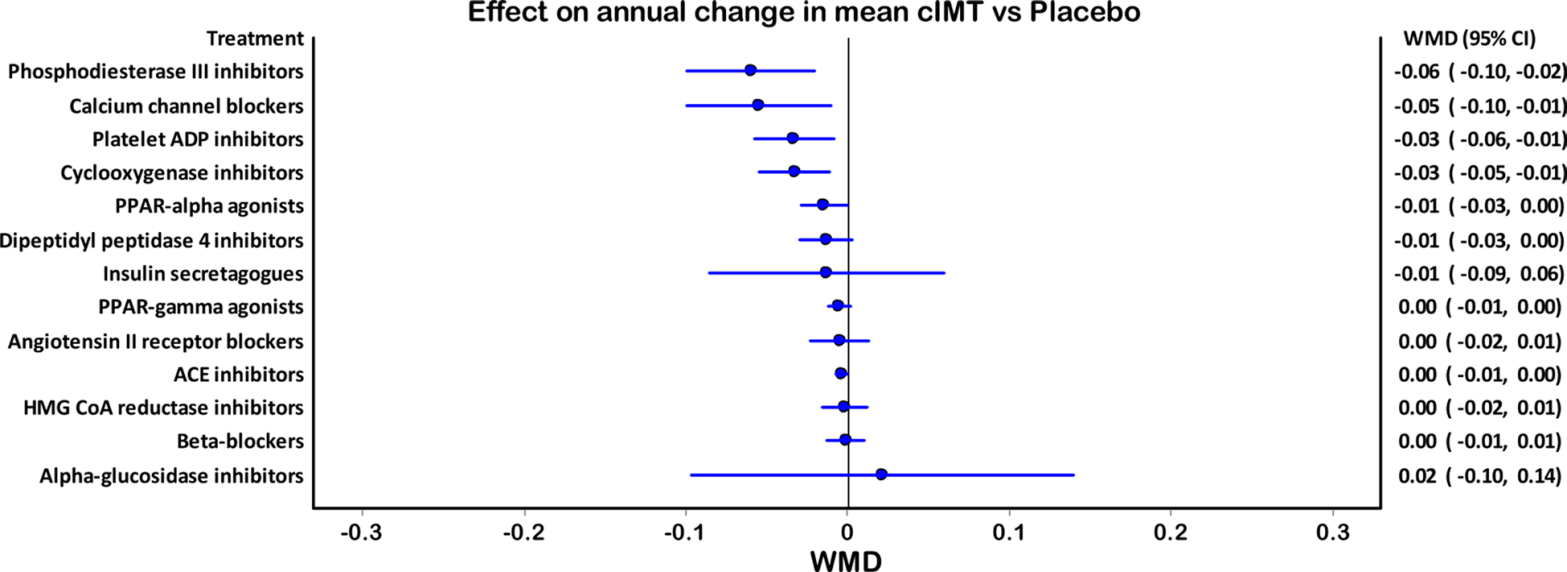
Forest graph showing the outcomes of NMA (relative efficacies of various drug classes in reducing annual mean cIMT)

In the overall network (Fig. 2), 13 drug classes (Table S2) were subjected to direct and indirect comparisons. Treatment duration was 25.8 months [22.9, 28.7]. Phosphodiesterase III inhibitors were found to be the most effective in retarding annual mean cIMT against network placebo (weighted mean difference (WMD) − 0.059 mm [95% CI − 0.099, − 0.020]) followed by the calcium channel blockers (WMD − 0.055 mm [95% CI − 0.099, 0.001]), platelet ADP inhibitors (WMD − 0.033 mm [95% CI − 0.058, 0.008] and cyclooxygenase inhibitors (WMD − 0.033 mm [95% CI − 0.054, 0.011]; Fig. 3). The outcomes of direct and indirect comparisons are given in Table S3 and the outcomes of all systematic NMAs are presented in Additional file 1: Figure S1a–m.

**Fig. 4:**
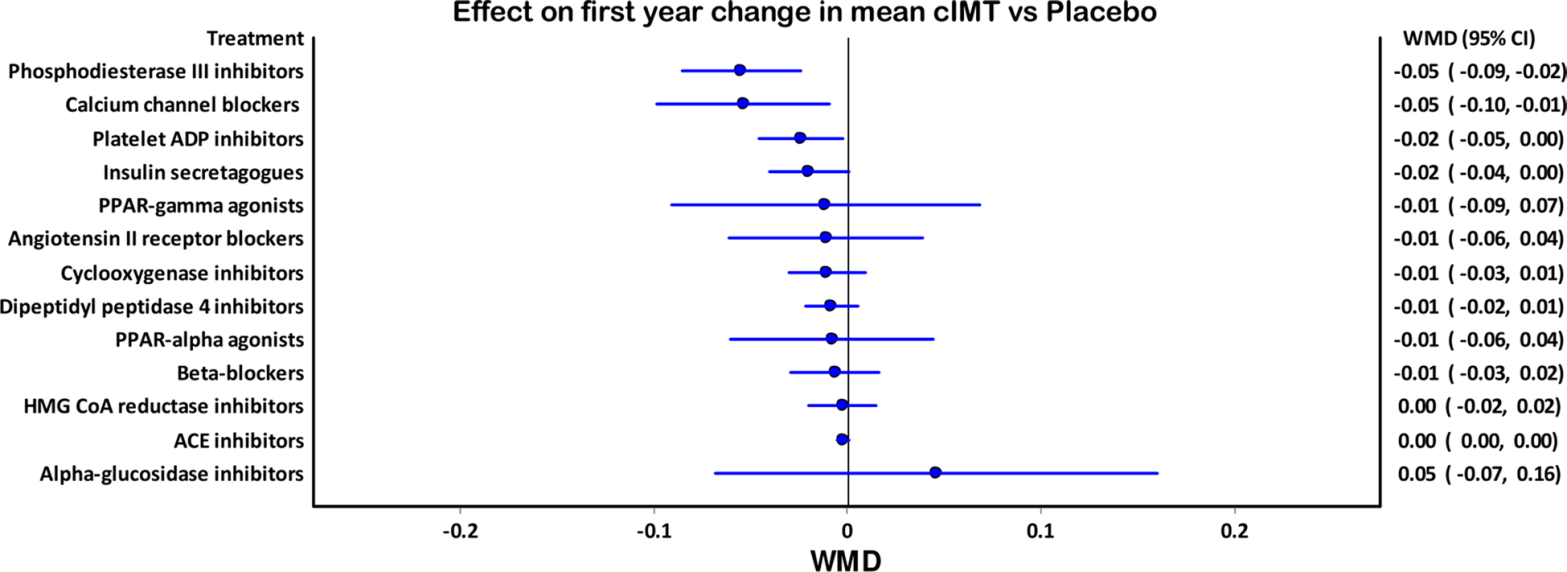
Forest graph showing the outcomes of a parallel NMA comparing the relative efficacies of various drug classes in reducing mean cIMT in the first year of treatment

In a parallel NMA, instead of annual changes, only first year changes in mean cIMT reported by the included studies were used; the outcomes were similar. In this NMA, Phosphodiesterase III inhibitors (WMD − 0.059 mm [95% CI − 0.099, − 0.0196]), calcium channel blockers (WMD − 0.055 mm [95% CI − 0.099, 0.0099]), platelet ADP inhibitors (WMD − 0.033 mm [95% CI − 0.058, 0.008], and cyclooxygenase inhibitors (WMD − 0.033 mm [95% CI − 0.058, 0.0084]) were found to be the most effective in retarding annual mean cIMT progression (Fig. 4).

### Sensitivity analyses

As a sensitivity analysis, a placebo-controlled network (no comparator in network) consisting of 10 classes studied in 36 trial arms was performed. In this network, treatment with phosphodiesterase III inhibitors (WMD − 0.051 mm [95% CI − 0.091, − 0.00]) was associated with the maximum reduction in annual mean cIMT, followed by the cyclooxygenase inhibitors (WMD − 0.03 mm [95% CI − 0.060, − 0.008]), and the platelet ADP inhibitors (WMD − 0.033 mm [95% CI − 0.064, − 0.002]; Additional file 1: Figure S2).

The outcomes of the placebo-controlled IV-het NMA were also compared with a placebo-controlled random-effects frequentist meta-analysis. In this meta-analysis, the phosphodiesterase III inhibitors (WMD − 0.05 mm [95% CI − 0.10, 0.00]; p = 0.05), platelet ADP inhibitors (WMD − 0.03 mm [95% CI − 0.06, − 0.00]; p = 0.04), PPAR-alpha agonists (WMD − 0.01 mm [95% CI − 0.03, 0.00]; p = 0.05), and PPAR-gamma agonists (WMD − 0.01 mm [95% CI − 0.01, − 0.00]; p = 0.008) showed significant reductions in annual change in mean cIMT (Additional file 1: Figure S3).

### Quality of evidence

With some caveats discussed in the limitation section, the quality of the evidence generated herein is moderate based on the following observations. Except for 2 prospective non-randomized trials, all the included studies were randomized and controlled and were of moderate (mainly due to blinding constraints) to high quality (Additional file 1: Table S4). Although, the Begg’s test showed no significant publication (adjusted Kendall’s Score − 157 ± 105.62 (SD); p = 0.137; Additional file 1: Figure S4), weightage could be affected by the imbalance in the number of included studies for different drug classes.

### Transitivity analyses

To determine the possible impact of modifiers on the NMA outcome, associations of the outcome (annual change in mean cIMT in treated and placebo groups) with important explanatory variables were estimated using metaregression (Additional file 1: Table S5). In the treatment group (any drug class), longer treatment duration, higher BMI, and a higher baseline cIMT were found to be independently associated with a lesser reduction in annual mean cIMT. However, in multivariate analysis with these 3 modifiers, none of these variables was significantly associated with the change in mean cIMT per annum. In placebo group, only increasing age was independently associated with a lesser reduction in mean cIMT per year. In correlation analyses, age was positively associated with baseline mean cIMT in both treatment and placebo groups (Additional file 1: Table S5).

## Discussion

Metabolic diseases can increase the risk of heart disease, stroke and diabetes. Therefore, medication is required not only to control active disease but also to reduce risk of possible future cardiovascular events. In this NMA, we compared the effects of 13 drug classes on the progression of cIMT and found that phosphodiesterase III inhibitors and calcium channel blockers were more effective than other therapies, although most drug classes were associated with retardation of cIMT progression. Age, baseline cIMT and BMI were identified as potential modifiers affecting the change.

A previous meta-analysis of 5 RCTs found that cilostazol, a selective phosphodiesterase III inhibitor, significantly reduced the progression of cIMT (WMD: − 0.08 mm [95% CI − 0.13, − 0.04; P = 0.00003) in a patient population of which 85% had type 2 diabetes mellitus [58], which is comparable to the annual change observed for this drug (WMD: − 0.059 mm [95% CI − 0.099, − 0.020]) in the present NMA. Phosphodiesterase III inhibitors may attenuate atherosclerosis by increasing cyclic adenosine monophosphate (cAMP) levels in platelets, vascular smooth cells and endothelial cells which then inhibit smooth muscle cell migration and proliferation [12]. Cilostazol is reported to inhibit high glucose or angiotensin II-induced proliferation of human vascular smooth muscle cells by inhibiting cell cycle transcription factor, E2F, or type 1 plasminogen activator inhibitor expression, in vitro [59, 60]. Cilostazol also decreases serum triglyceride and LDL-cholesterol levels, increases HDL-cholesterol levels, improves insulin sensitivity, increases nitric oxide production, prevents production of adhesion molecules, and attenuates endothelial dysfunction [28].

Calcium channel blockers may also reduce cIMT progression by several mechanisms. For example, amlodipine has been reported to inhibit the growth and migration of vascular smooth muscles [61] and to manifest antioxidant effects to retard atherosclerosis [62]. Lacidipine, in addition to exerting antioxidant effects, also reduces adhesion molecule expression in endothelial cells and restores endothelium dependent vasodilatation in essential hypertensive patients [63, 64]. In a review of RCTs, calcium-channel blockers were found to be more effective than other antihypertensive drugs including diuretics, beta-blockers, and ACE inhibitors in blunting cIMT progression [65].

Previous reviews have also found that statins [66], and alpha-glucosidase inhibitors [67] are capable of attenuating cIMT progression. Statins may reverse cIMT by reducing lipids, inflammation, and oxidative stress, and they may change the histological characteristics of plaque [68]. Improved insulin sensitivity may play a role in atheroprotection [69] and an increase in HDL-cholesterol levels [69] and reduction in LDL-cholesterol [70] are associated with reduction of cIMT progression.

To date, less data are available for GLP-1 agonists, sodium-chloride transporter inhibitors, and SGLT2 inhibitors in association with cIMT progression. A meta-analysis of 5 studies found that 3–12 months of GLP-1 based therapies decreased cIMT, although statistically non-significantly [71]. A prospective cohort study of 35 type 2 diabetes outpatients found significantly reduced IMT in the empagliflozin and liraglutide-treated patients after 3 months of treatment [72]. Sitagliptin and liraglutide treatment also improved arterial stiffness, diastolic function and myocardial strain by reducing oxidative stress in subjects with newly diagnosed type 2 diabetes [73, 74].

In diabetes patients, atherosclerosis is a leading cause of mortality due to cardiovascular or cerebrovascular disease or sudden death [75]. CVD is 2–4 times more prevalent in diabetes patients than in the normal population. The mean difference in IMT observed between diabetes patients and non-diabetes individuals is 0.05–0.08 mm [76]. Glycosylated hemoglobin and the duration of diabetes have also been found to be positively associated with cIMT [77, 78]. For these reasons, prevention or retardation of atherosclerosis is more important in diabetes patients.

In the transitivity analyses, we also observed that a higher BMI was independently associated with a lesser reduction in annual mean cIMT. In a community-based study of 676 individuals, BMI was found to be a significant predictor of progression of cIMT in black but not white individuals after controlling for age, race, sex, and traditional CVD risk factors [79]. Moreover, BMI has been found to have a significantly positive correlation with cIMT [80, 81].

Some limitations of the present study should to be considered. An important limitation was the unavailability of an adequate number of studies for some drug classes such as sodium-chloride transporter inhibitors, SGLT2 inhibitors and GLP-1 receptor agonists. Another factor possibly affecting the NMA outcomes could be the varying number of studies for a drug class in the network as we had more data available for some classes such as PPAR-gamma agonists and statins but less for others such as platelet ADP inhibitors and cyclooxygenase inhibitors. Another shortcoming could arise from the grouping of PPAR-gamma agonists because pioglitazone and rosiglitazone are reported to have contrasting effects on cardiovascular outcomes. However, a sub-analysis of the present study showed were no significant differences between these 2 drugs in reducing cIMT.

Results of this study indicate that cardiovascular risk factors play a role in the progression of cIMT, and the drug environment may modify this process, reiterating the importance of timely therapeutic interventions in the control of cardiovascular risk factors. Because atherosclerosis is a progressive process and is affected by several risk factors, reduction in cIMT or slowing its progression can lead to the prevention or delaying of CVD events. Our finding (which is mainly based on primary prevention studies) that drugs, especially the phosphodiesterase III inhibitors and calcium channel blockers, significantly retard cIMT progression indicates that medication can significantly influence the relationship between IMT progression and CVD risk. We have also observed that a greater reduction in cIMT occurred in patients with high cIMT values at baseline. Thus, earlier treatment can improve the chances of a better prognosis in the long run. As Rundek et al. pointed out that the presence of plaque may be an important confounder in the predictive models of IMT, therefore a separate characterization of plaque and IMT is necessary for vascular disease risk assessment [82]. Our results identify medication use as another confounder in the predictive models of IMT progression and vascular disease risk assessment.

## Conclusion

In this NMA, 13 drug classes used for the management of metabolic disorders (antihypertensive, antiplatelet, hypoglycemic, and lipid lowering) studied in 47 trials involving 15,721 subjects treated for approximately 2 years (25.8 months [95% CI 22.9, 28.7]), phosphodiesterase III inhibitors and calcium channel blockers were found more effective than others in retarding cIMT per annum. Age, BMI, and baseline cIMT may have some impact on these outcomes. Currently, less data are available for platelet ADP inhibitors, GLP-1 receptor agonists, sodium-chloride transporter inhibitors, and SGLT2 inhibitors.

## Additional file

**Additional file 1.** Additional figures and tables.

## Abbreviations

ADDIS: Aggregate Data Drug Information System
ADP: adenosine diphosphate
BMI: body mass index
cIMT: carotid artery intima-media thickness
CVD: cardiovascular disease
GRADE: Grading of Recommendations Assessment, Development and Evaluation
GLP-1: glucagon like peptide-1
GPM: Generalized Pairwise Modeling
HDL: high-density lipoprotein
HMG CoA: hydroxy-methylglutaryl coenzyme A
IMT: intima-media thickness
IVhet: inverse variance-heterogeneity
LDL: low-density lipoproteins
MCMC: Markov Chain Monte Carlo
NMA: network meta-analysis
PPAR: peroxisome proliferator-activated receptor
PRISMA: Preferred Reporting Items for Systematic Reviews and Meta-analyses
RCTs: randomized controlled trials
SGLT2: sodium-glucose cotransporter-2
