# Supplementary material for: Comparative effects of lipid lowering, hypoglycemic, antihypertensive and antiplatelet medications on carotid artery intima-media thickness progression: a network meta-analysis
Source: Cardiovasc Diabetol. 2019 Jan 30;18:14. doi: 10.1186/s12933-019-0817-1 (PMC6352423; doi:10.1186/s12933-019-0817-1)
Supplement: Supplementary file 1 — Additional file 1. Additional figures and tables. [file 12933_2019_817_MOESM1_ESM.docx]

| Table S1: Important characteristics of the included studies | | | | | | | | | | | | | | | | |  |
| --- | --- | --- | --- | --- | --- | --- | --- | --- | --- | --- | --- | --- | --- | --- | --- | --- | --- |
| Study ID [Ref] | **Trial ID** | **nT** | **nP** | **nC** | **TD** | **Condition** | **Drug** | **Comparator** | **Design** | **Age (years)** | **% males** | **Disease years** | **BMI** | **% smokers** | **% CVD** |  |  |
| Ahn 2001 [11] |  | 60 | 60 |  | 12 | Type 2 Diabetes Mellitus | Cilostazol | Placebo | SB-RCT | 62±7 | 50 | 9.8±6.3 | 23.3±3 | 24 | 5 |  |  |
| Ahn 2011 [12] |  | 54 | 66 |  | 24 | Acute coronary syndrome | Cilostazol | Placebo | SB-RCT | 63.6±11 | 62 |  | 24.3±4 | 45 |  |  |  |
| Arrif 2006 [13] |  | 44 |  | 44 | 12 | Hypertension | Candesartan | Atenolol | DB-RCT | 55±8 | 63 | 3.5±12 |  | 8 |  |  |  |
| Asselbergs 2005 [14] | PREVEND | 319 | 323 |  | 48 | Albuminuria | Fosinopril | Pravastatin | DB-RCT | 50.8±11 | 65 |  |  |  | 3 |  |  |
| Baguet 2009 [15] | MITEC | 100 |  | 109 | 36 | Type 2 Diabetes Mellitus | Candesartan | Amlodipine | DB-RCT | 59.7±9 | 60 |  | 31±6 |  |  |  |  |
| Beishuizen 2004 [16] |  | 125 | 125 |  | 24 | Type 2 Diabetes Mellitus | Simvastatin | Placebo | DB-RCT | 58.5±11 | 47.5 | 6.5±7.5 | 31±6 | 24 |  |  |  |
| Bots 2009 [17] | METEOR | 377 | 375 |  | 24 | Hypercholesteremia | Rosuvastatin | Placebo | DB-RCT | 57±6 | 59.5 |  | 27.3±4 | 4.5 |  |  |  |
| de Groot 1995 [18] | REGRESS | 450 | 434 |  | 24 | Coronary artery disease | Pravastatin | Placebo | DB-RCT | 56.2±8 |  |  | 26±2.7 | 28 | 100 |  |  |
| Elkeles 1998 [19] | SENDCAP | 81 | 83 |  | 36 | Type 2 Diabetes Mellitus | Bezafibrate | Placebo | DB-RCT | 50.9±8 | 71 | 5±5 | 28.7±5 |  |  |  |  |
| Hanefeld 2004 [20] | STOP-NIDDM | 56 | 59 |  | 48 | Impaired glucose tolerance | Acarbose | Placebo | DB-RCT | 55.3±7 | 61.3 |  | 29±4 | 27.2 |  |  |  |
| Hedbald 2001 [21] | BCAPS | 199 |  | 198 | 36 | Asymptomatic atherosclerosis | Metoprolol | Fluvastatin | DB-RCT | 61.5±5 | 53.5 |  | 25.6±4 | 30 |  |  |  |
| Hedbald 2007 [22] |  | 81 | 84 |  | 12 | Type 2 Diabetes Mellitus | Rosiglitazone | Placebo | DB-RCT | 66.5±7 | 55 |  | 29.5±5 | 14.5 | 7.5 |  |  |
| Hiukka 2016 [23] | FIELD | 83 | 87 |  | 60 | Type 2 Diabetes Mellitus | Fenofibrate | Placebo | DB-RCT | 62±7 |  | 5.5±2 | 29.4±8 | 14.5 | 29 |  |  |
| Hodis 2006 [24] | TART | 142 | 134 |  | 24 | Type 2 Diabetes Mellitus | Troglitazone | Placebo | DB-RCT | 52.5±9 | 32.5 | 9.8±6.3 | 31.8±6 | 66 |  |  |  |
| Hosomi 2001 [25] |  | 48 | 50 |  | 24 | Type 2 Diabetes Mellitus | Enallapril | Placebo | OL-RCT | 56.4±9 | 62 |  | 23.6±3 | 45.2 |  |  |  |
| Igase 2012 [26] |  | 26 | 25 |  | 12 | Dyslipidemia | Rosuvastatin | Placebo | SB-RCT | 66.9±6 | 0 |  | 23.3±3 |  |  |  |  |
| Ishikawa 2014 [27] | UMIN 000006432 | 37 | 39 |  | 12 | Impaired glucose tolerance | Sitagliptin | Placebo | OL-RCT | 71.4±8 | 85.2 |  | 24.6±4 |  |  |  |  |
| Katakami 2010 [28] | DAPC | 145 |  | 152 | 24 | Type 2 Diabetes Mellitus | Cilostazol | Aspirin | OL-RCT | 62.5±8 | 52 | 12.8±8.5 | 24.5±4 | 30.5 |  |  |  |
| Kodama 2000 [29] |  | 34 | 40 | 40 | 36 | Type 2 Diabetes Mellitus | Ticlopidine | Aspirin | OL-RCT | 65.2±1 | 55 | 19±1.5 |  |  |  |  |  |
| Koshiyama 1999 [30] |  | 11 |  | 11 | 6 | Type 2 Diabetes Mellitus | Amlodipine | ACE inhibitors | Prospective |  |  |  |  |  |  |  |  |
| Koshiyama 2001 [31] |  | 56 |  | 56 | 6 | Type 2 Diabetes Mellitus | Pioglitazone | Sulfonylurea | OL-RCT | 62±3.6 | 57 |  |  |  |  |  |  |
| Koyasu 2010 [32] | UMIN 00000544 | 42 | 39 |  | 12 | Impaired glucose tolerance | Acarbose | Placebo | OL-RCT | 66.3±8 | 91.7 |  | 24.7±3 | 6.1 |  |  |  |
| Langenfeld 2005 [33] |  | 89 |  | 84 | 6 | Type 2 Diabetes Mellitus | Pioglitazone | Glimepiride | OL-RCT | 62.5±8 | 61.9 | 7.2±6.7 |  |  |  |  |  |
| Lonn 2009 [34] | STARR | 681 | 666 |  | 36 | Impaired glucose tolerance | Ramipril | Rosiglitazone | DB-RCT | 54.3±11 | 45 |  | 30.2±5 |  |  |  |  |
| Ludwig 2002 [35] | LAARS | 142 |  | 138 | 24 | Hypertension | Losartan | Atenolol | DB-RCT | 58.8±8.7 | 50 |  | 28±4 |  |  |  |  |
| Mazzone 2016 [36] | CHICAGO | 175 |  | 186 | 18 | Type 2 Diabetes Mellitus | Plioglitazone | Glimepiride | DB-RCT | 59.6±8 | 63.5 | 7.4±7 | 32±5 |  |  |  |  |
| Minamikawa 1998 [37] |  | 57 |  | 78 | 6 | Type 2 Diabetes Mellitus | Troglitazone | Sulfonylurea | Prospective | 61.5±10.5 | 65 |  |  |  |  |  |  |
| Mita 2007 [38] |  | 34 | 36 |  | 12 | Type 2 Diabetes Mellitus | Nateglinide | Placebo | OL-RCT | 61.5±7 | 47 | 4.6±2.8 | 23.6±3 | 71 |  |  |  |
| Mita 2015 [39] | SPEAD-A | 172 | 169 |  | 24 | Type 2 Diabetes Mellitus | Alogliptin | Placebo | OL-RCT | 64.6±9.5 | 62 | 8.7±1.4 |  | 24 | 0 |  |  |
| Mita 2016 [40] | SPIKE | 142 | 140 |  | 24 | Type 2 Diabetes Mellitus | Sitagliptin | Placebo | OL-RCT | 63.7±9.8 | 60.5 | 17.3±8.6 |  | 21.5 |  |  |  |
| Mitsuhashi 2004 [41] |  | 31 | 31 |  | 32 | Type 2 Diabetes Mellitus | Cilostazol | Placebo | OL-RCT | 63.5±7.6 |  | 64.5±9 | 23.5±8 |  |  |  |  |
| Mortsell 2007 [42] | SILVHIA | 52 |  | 56 | 12 | Hypertension | Irbesartan | Atenolol | DB-RCT | 54±9 | 67 |  | 27±3 |  |  |  |  |
| Nakamura 2004 [43] |  | 15 |  | 15 | 12 | Type 2 Diabetes Mellitus | Pioglitazone | Gilbenclamide / Vogliobose | OL-RCT | 55.5±12 | 56 | 17±4.8 |  |  |  |  |  |
| Ohta 2013 [44] | HOSP | 37 |  | 43 | 60 | Hypertension | Amlodipine | Losartan | SB-RCT | 64±9 | 49 |  | 28.5±2.7 |  |  |  |  |
| Olsen 2005 [45] | LIFE | 22 |  | 23 | 36 | Hypertension | Atenolol | Losartan | OL-RCT | 65±6 | 77 |  |  |  |  |  |  |
| Oyama 2008 [46] |  | 31 | 21 |  | 12 | Type 2 Diabetes Mellitus | Acarbose | Placebo | OL-RCT | 60±5 | 44 | 12±6.8 | 23.1±3 |  |  |  |  |
| Oyama 2016 [47] | PROLOGUE | 222 | 220 |  | 24 | Type 2 Diabetes Mellitus | Sitagliptin | Placebo | OL-RCT | 69.3±9 | 67 |  | 25.2±4 |  | 8 |  |  |
| Patel 2013 [48] | EDIP | 109 | 110 |  | 60 | Impaired glucose tolerance | Acarbose | Placebo | OL-RCT | 53.6±11 | 34 |  | 35.3±7 |  |  |  |  |
| Sawayama 2002 [49] | FAST | 81 | 81 |  | 24 | Hypercholesteremia | Pravastatin | Placebo | OL-RCT | 66.1±14 | 77 |  | 23.2±4 | 59.3 | 14.2 |  |  |
| Shinoda-Tagawa 2002 [50] |  | 43 | 46 |  | 38 | Type 2 Diabetes Mellitus | Cilostazol | Placebo | SB-RCT | 61±8 | 40 | 11.5±10 | 23±4 |  |  |  |  |
| Sidhu 2004 [51] |  | 46 | 46 | 46 | 12 | Coronary artery disease | Rosiglitazone | Placebo | DB-RCT | 62.5±8 | 86 |  | 27±4 |  | 100 |  |  |
| Stanton 2001 [52] |  | 35 |  | 34 | 50 | Hypertension | Amlodipine | Lisinopril | DB-RCT | 49±12 | 59 |  |  | 34 |  |  |  |
| Stumpe 2007 [53] | MORE | 78 |  | 76 | 24 | Hypertension | Olmesartan | Atenolol | DB-RCT | 62.2±7 | 61 | 8.8±7.1 | 27.4±3 |  | 11.5 |  |  |
| Wiklund 2002 [54] |  | 40 | 52 |  | 36 | Hypercholesteremia | Metoprolol | Placebo | DB-RCT | 60±10 | 51 |  | 24.8±3 | 27 |  |  |  |
| Xiang 2005 [55] | TRIPOD | 93 | 99 |  | 36 | Impaired glucose tolerance | Troglitazone | Placebo | DB-RCT | 34.6±6.8 |  |  | 30±5 |  |  |  |  |
| Yamamoto 2010 [56] |  | 29 |  | 28 | 18 | Type 2 Diabetes Mellitus | Losartan | Amlodipine | SB-RCT | 61±11 | 80 |  |  |  |  |  |  |
| Yasunari 2010 [57] |  | 22 | 26 |  | 12 | Type 2 Diabetes Mellitus | Pioglitazone | Placebo | SB-RCT | 56.7±10.4 | 75 | 14.1±7.3 | 26.4±3.7 |  |  |  |  |
| Abbreviations: AA, asymptomatic atherosclerosis; ACS, acute coronary syndrome; ALB, albuminuria; BMI, body mass index; CAD, coronary artery disease; CVD, cardiovascular disease; DB-RCT, double-blind randomized controlled trial; DD, disease duration (years); HC, hypercholesterolemia; HTN, hypertension; IGT, impaired glucose tolerance; nC/nP/nT, number of patients in comparator/placebo/treatment group; OL-RCT, open-label RCT; SB-RCT, single-blind RCT; sd, standard deviation; TD, treatment duration; T2DM, type 2 diabetes mellitus; .  Trial nomenclature: BCAPS - Beta-Blocker Cholesterol-Lowering Asymptomatic Plaque Study; CAIUS - The Carotid Atherosclerosis Italian Ultrasound Study; CHICAGO - Carotid Intima-Media Thickness in Atherosclerosis Using Pioglitazone; DAPC - Diabetic Atherosclerosis Prevention by Cilostazol Study; EDIP - Early Diabetes Intervention Program; ELSA, European Lacidipine Study on Atherosclerosis; FAST - Fukuoka Atherosclerosis Trial; FIELD - Fenofibrate Intervention and Event Lowering in Diabetes; HOSP, Home Systolic Pressure; J-ELAN, Japanese Patients with Mild-to-Moderate Hypertension; LAARS, The Losartan Vascular Regression Study; LIFE, Losartan Intervention For Endpoint-reduction in hypertension; METEOR - Measuring Effects on intima media Thickness: an Evaluation Of Rosuvastatin; MITEC - Media Intima Thickness Evaluation with Candesartan cilexetil; MORE - Multicenter Olmesartan atherosclerosis Regression Evaluation study; PREVEND - Prevention of REnal and Vascular ENdstage Disease Intervention Trial; PROLOGUE, Program of Vascular Evaluation under Glucose Control by DPP-4 Inhibitor; SILVHIA - Swedish Irbesartan Left Ventricular Hypertrophy Investigation versus Atenolol; SPEAD-A; SPIKE; STARR, STudy of Atherosclerosis with Ramipril and Rosiglitazone; STOP-NIDDM; TART - Troglitazone Atherosclerosis Regression Trial; UMIN 000006432; UMIN000000544. | | | | | | | | | | | | | | | | | |

| Table S2: Therapeutic agents used in the included studies | |
| --- | --- |
| Class | **Drugs in the network** |
| Alpha-glucosidase inhibitors | Acarbose (4), Voglibose (1) |
| Angiotensin converting enzyme inhibitors | Ramipril (2), Fosinopril, Enallapril, lisinopril (1 each) |
| Angiotensin II receptor blockers | Losartan (4), Candesartan (2), Irbesartan, and Olmesartan medoxomil (1 each) |
| Beta-blockers | Atenolol (5), Metoprolol (3) |
| Calcium channel antagonists | Amlodipine (5) |
| Cyclooxygenase inhibitors | Aspirin (2) |
| Dipeptidyl peptidase-4 inhibitors | Sitagliptin (3), Alogliptin (1) |
| Hydroxy-methylglutaryl coenzyme A reductase inhibitors | Pravastatin (3), Fluvastatin (2), Rosuvastatin (2), Simvastatin (1) |
| Insulin secretagogues | Glimepiride (3), Nateglinide (2), Gilbenclamide (2) |
| Phosphodiesterase III inhibitors | Cilostazol (5) |
| Peroxisome proliferator-activated receptor alpha agonists | Bezafibrate (1), Fenofibrate (1) |
| Peroxisome proliferator-activated receptor gamma agonists | Pioglitazone (6), Rosiglitazone (4), Troglitazone (3) |
| Platelet ADP inhibitors | Ticlopidine (1) |

| Table S3: Direct and indirect comparison in the NMA | | | | | |  |
| --- | --- | --- | --- | --- | --- | --- |
| Direct estimates | | | | | |  |
| ID | Active | Control | WMD | LCI 95% | HCI 95% |  |
| 1 | Alpha-glucosidase inhibitors | Placebo | -0.006 | -0.0725 | 0.060284 |  |
| 2 | ACE inhibitors | Placebo | -0.003 | -0.00958 | 0.003509 |  |
| 3 | Beta-blockers | Angiotensin II receptor blockers | 0.002 | -0.01093 | 0.015441 |  |
| 4 | Calcium channel blockers | Angiotensin II receptor blockers | -0.006 | -0.04299 | 0.031703 |  |
| 5 | Calcium channel blockers | ACE inhibitors | -0.052 | -0.0957 | -0.00742 |  |
| 6 | ACE inhibitors | HMG CoA reductase inhibitors | -0.002 | -0.02692 | 0.02282 |  |
| 7 | ACE inhibitors | PPAR-gamma agonists | 0.001 | -0.00211 | 0.004506 |  |
| 8 | Beta-blockers | Placebo | -0.002 | -0.01499 | 0.010394 |  |
| 9 | Beta-blockers | HMG CoA reductase inhibitors | 0.004 | -0.01365 | 0.020915 |  |
| 10 | Cyclooxygenase inhibitors | Placebo | -0.034 | -0.06025 | -0.00775 |  |
| 11 | Dipeptidyl peptidase 4 inhibitors | Placebo | -0.013 | -0.02915 | 0.003231 |  |
| 12 | HMG CoA reductase inhibitors | Placebo | 0.005 | -0.02303 | 0.032416 |  |
| 13 | Insulin secretagogues | Placebo | -0.041 | -0.10799 | 0.02599 |  |
| 14 | Platelet ADP inhibitors | Placebo | -0.033 | -0.06371 | -0.00229 |  |
| 15 | Platelet ADP inhibitors | Cyclooxygenase inhibitors | 0.001 | -0.03089 | 0.032886 |  |
| 16 | Phosphodiesterase III inhibitors | Placebo | -0.052 | -0.10415 | 0.000388 |  |
| 17 | Phosphodiesterase III inhibitors | Cyclooxygenase inhibitors | -0.036 | -0.09046 | 0.019456 |  |
| 18 | PPAR-alpha agonists | Placebo | -0.014 | -0.02825 | 3.45E-05 |  |
| 19 | PPAR-gamma agonists | Placebo | -0.005 | -0.00816 | -0.00171 |  |
| 20 | PPAR-gamma agonists | Insulin secretagogues | -0.038 | -0.12318 | 0.04672 |  |
| 21 | PPAR-gamma agonists | Alpha-glucosidase inhibitors | -0.115 | -0.2328 | 0.002798 |  |
|  |  |  |  |  |  |  |
| Indirect estimates (source IDs) | | | | | | |
| 22 | Indirect Calcium channel blockers vs Placebo (5, 2) | | -0.055 | -0.09922 | -0.00997 | |
| 23 | Indirect ACE inhibitors vs Placebo (6, 12) | | 0.003 | -0.0346 | 0.039886 | |
| 24 | Indirect ACE inhibitors vs Placebo (7, 19) | | -0.004 | -0.00835 | 0.000888 | |
| 25 | Indirect Beta-blockers vs Placebo (9, 12) | | 0.008 | -0.02435 | 0.040994 | |
| 26 | Indirect Platelet ADP inhibitors vs Placebo (15, 10) | | -0.033 | -0.0743 | 0.0083 | |
| 27 | Indirect Phosphodiesterase III inhibitors vs Placebo (17, 10) | | -0.070 | -0.1304 | -0.0086 | |
| 28 | Indirect PPAR-gamma agonists vs Placebo (20, 13) | | -0.079 | -0.18742 | 0.028956 | |
| 29 | Indirect PPAR-gamma agonists vs Placebo (21, 1) | | -0.121 | -0.25633 | 0.014111 | |
| 30 | Indirect Angiotensin II receptor blockers vs Placebo (3, 8) | | -0.005 | -0.02286 | 0.013749 | |
| 31 | Indirect HMG CoA reductase inhibitors vs Placebo (6, 2) | | -0.001 | -0.027 | 0.024732 | |
| 32 | Indirect PPAR-gamma agonists vs Placebo (7, 2) | | -0.004 | -0.012 | 0.003097 | |
| 33 | Indirect HMG CoA reductase inhibitors vs Placebo (9, 8) | | -0.006 | -0.027 | 0.015509 | |
| 34 | Indirect Cyclooxygenase inhibitors vs Placebo (15, 14) | | -0.034 | -0.078 | 0.010272 | |
| 35 | Indirect Cyclooxygenase inhibitors vs Placebo (17, 16) | | -0.016 | -0.092 | 0.059463 | |
| 36 | Indirect Insulin secretagogues vs Placebo (20, 19) | | 0.033 | -0.052 | 0.118315 | |
| 37 | Indirect Alpha-glucosidase inhibitors vs Placebo (21, 19) | | 0.110 | -0.008 | 0.227911 | |
|  |  | |  |  |  | |
| Result estimates (source IDs) | | | | | | |
|  | Alpha-glucosidase inhibitors (1, 37) | | 0.022 | -0.096 | 0.140147 | |
|  | ACE inhibitors (2, 23, 24) | | -0.003 | -0.007 | 0.000317 | |
|  | Beta-blockers (8, 25) | | -0.001 | -0.013 | 0.010926 | |
|  | Angiotensin II receptor blockers (30) | | -0.005 | -0.023 | 0.013749 | |
|  | Calcium channel blockers (22) | | -0.055 | -0.099 | -0.00997 | |
|  | HMG CoA reductase inhibitors (12, 31, 33) | | -0.002 | -0.016 | 0.012497 | |
|  | PPAR-gamma agonists (19, 28, 29, 32) | | -0.005 | -0.012 | 0.001971 | |
|  | Cyclooxygenase inhibitors (10, 34, 35) | | -0.033 | -0.054 | -0.01093 | |
|  | Dipeptidyl peptidase 4 inhibitors (11) | | -0.013 | -0.029 | 0.003231 | |
|  | Insulin secretagogues (13, 36) | | -0.013 | -0.085 | 0.060067 | |
|  | Platelet ADP inhibitors (14, 26) | | -0.033 | -0.058 | -0.00835 | |
|  | Phosphodiesterase III inhibitors (16, 27) | | -0.059 | -0.099 | -0.01969 | |
|  | PPAR-alpha agonists (18) | | -0.014 | -0.028 | 3.45E-05 | |

| Table S4: Quality Assessment of the Included Studies | | | | | | | |
| --- | --- | --- | --- | --- | --- | --- | --- |
| Study | **Other bias** | **Selective reporting** | **Incomplete outcome data** | **Blinding of outcome assessment** | **Blinding of participants / personnel** | **Allocation concealment** | **Random sequence generator** |
| Ahn 2001 [11] | L | L | L | U | H | L | L |
| Ahn 2011 [12] | L | L | L | L | H | L | L |
| Arrif 2006 [13] | L | L | L | L | L | L | L |
| Asselbergs 2005 [14] | L | L | L | L | L | L | L |
| Baguet 2009 [15] | L | L | L | L | L | L | L |
| Beishuizen 2004 [16] | L | L | L | L | L | L | L |
| Bots 2009 [17] | L | L | L | L | L | L | L |
| de Groot 1995 [18] | L | L | L | L | L | L | L |
| Elkeles 1998 [19] | L | L | L | L | L | L | L |
| Hanefeld 2004 [20] | L | L | L | L | L | L | L |
| Hedbald 2001 [21] | L | L | L | L | L | L | L |
| Hedbald 2007 [22] | L | L | L | L | L | L | L |
| Hiukka 2016 [23] | L | L | L | L | L | L | L |
| Hodis 2006 [24] | L | L | L | L | L | L | L |
| Hosomi 2001 [25] | L | L | L | U | H | L | L |
| Igase 2012 [26] | L | L | L | L | H | L | L |
| Ishikawa 2014 [27] | L | L | L | U | H | L | L |
| Katakami 2010 [28] | L | L | L | L | H | L | L |
| Kodama 2000 [29] | L | L | L | U | H | L | L |
| Koshiyama 1999 [30] | L | L | L | U | H | L | L |
| Koshiyama 2001 [31] | L | L | L | U | H | H | H |
| Koyasu 2010 [32] | L | L | L | L | H | L | L |
| Langenfeld 2005 [33] | L | L | L | L | H | L | L |
| Lonn 2009 [34] | L | L | L | L | L | L | L |
| Ludwig 2002 [35] | L | L | L | L | L | L | L |
| Mazzone 2016 [36] | L | L | L | L | L | L | L |
| Minamikawa 1998 [37] | L | L | L | U | H | H | H |
| Mita 2007 [38] | L | L | L | L | H | L | L |
| Mita 2015 [39] | L | L | L | L | H | L | L |
| Mita 2016 [40] | L | L | L | L | H | L | L |
| Mitsuhashi 2004 [41] | L | L | L | U | H | L | L |
| Mortsell 2007 [42] | L | L | L | L | L | L | L |
| Nakamura 2004 [43] | L | L | L | U | H | L | L |
| Ohta 2013 [44] | L | L | L | U | H | L | L |
| Olsen 2005 [45] | L | L | L | U | H | L | L |
| Oyama 2008 [46] | L | L | L | L | H | L | L |
| Oyama 2016 [47] | L | L | L | L | H | L | L |
| Patel 2013 [48] | L | L | L | L | H | L | L |
| Sawayama 2002 [49] | L | L | L | U | H | L | L |
| Shinoda-Tagawa 2002 [50] | L | L | L | L | H | L | L |
| Sidhu 2004 [51] | L | L | L | L | L | L | L |
| Stanton 2001 [52] | L | L | L | L | L | L | L |
| Stumpe 2007 [53] | L | L | L | L | L | L | L |
| Wiklund 2002 [54] | L | L | L | L | L | L | L |
| Xiang 2005 [55] | L | L | L | L | L | L | L |
| Yamamoto 2010 [56] | L | L | L | L | H | L | L |
| Yasunari 2010 [57] | L | L | L | H | H | L | L |
| Legends: H, high; L, low; U, unclear | | | | | | | |

| Table S5: Transitivity analyses – effect of modifiers on the outcomes of NMA  Treatment group | | | | | | |
| --- | --- | --- | --- | --- | --- | --- |
|  | **Relationship with annual change in mean cIMT** | | | **Correlation with baseline cIMT** | | |
| Modifier | **n** | **Metaregression coefficient [95% CI]** | **p** | **r** | **p** | **n** |
| Treatment duration | 74 | 0.0009 [0.00047, 0.00133] | **˂0.00001** | 0.079 | 0.534 | 74 |
| Age | 70 | −0.0002 [−0.00145, 0.0011] | 0.797 | 0.4579 | **0.0002** | 65 |
| Gender (% males) | 66 | 0.00012 [−0.0005, 0.0007] | 0.709 | 0.141 | 0.284 | 60 |
| Disease duration | 29 | 0.0006 [−0.0012, 0.0024] | 0.490 | 0.30 | 0.112 | 29 |
| Body mass index | 45 | 0.0023 [−0.00004, 0.0047] | **0.054** | −0.318 | **0.034** | 44 |
| Percent smokers | 26 | −0.00008 [−0.0007, 0.0005] | 0.767 | −0.050 | 0.815 | 24 |
| Baseline cIMT (mm) | 65 | 0.0417 [0.0182, 0.0653] | **0.001** |  |  |  |
| Placebo group | | | | | | |
|  | **Relationship with annual change in mean cIMT** | | | **Correlation with baseline cIMT** | | |
| Modifier | **n** | **Metaregression coefficient [95% CI]** | **p** | **r** | **p** | **n** |
| Treatment duration | 74 | 0.0001 [-0.00058, 0.00079] | 0.757 | 0.087 | 0.643 | 31 |
| Age | 70 | 0.0021 [0.0003, 0.0038] | **0.023** | 0.4781 | **0.0065** | 65 |
| Gender (% males) | 66 | 0.00035 [−0.00023, 0.0009] | 0.223 | 0.1124 | 0.569 | 31 |
| Disease duration | 29 | 0.00076 [−0.0014, 0.0029] | 0.456 | 0.306 | 0.309 | 13 |
| Body mass index | 45 | −0.0024 [−0.00597, 0.0013] | 0.192 | −0.2574 | 0.204 | 26 |
| Percent smokers | 26 | −0.00029 [−0.00137, 0.00079] | 0.579 | −0.125 | 0.621 | 18 |
| Baseline cIMT (mm) | 65 | 0.0932 [0.0553, 0.1311] | **˂0.00001** |  |  |  |
| Abbreviations: CVD, cardiovascular disease; n, number of study datasets; r, correlation coefficient; p, significance level | | | | | | |

**Figure S1a: Outcomes of the NMA with comparisons versus alpha-glucosidase inhibitors**

**Figure S1b: Outcomes of the NMA with comparisons versus ACE inhibitors**

**Figure S1c: Outcomes of the NMA with comparisons versus angiotensin II receptor blockers**

**Figure S1d: Outcomes of the NMA with comparisons versus beta-blockers**

**Figure S1e: Outcomes of the NMA with comparisons versus calcium channel blockers**

**Figure S1f: Outcomes of the NMA with comparisons versus cyclooxygenase inhibitors**

**Figure S1g: Outcomes of the NMA with comparisons versus dipeptidyl dipeptidase 4 inhibitors**

**Figure S1h: Outcomes of the NMA with comparisons versus HMG CoA reductase inhibitors**

**Figure S1i: Outcomes of the NMA with comparisons versus insulin secretagogues**

**Figure S1j: Outcomes of the NMA with comparisons versus phosphodiesterase III inhibitors**

**Figure S1k: Outcomes of the NMA with comparisons versus PPAR-alpha agonists**

**Figure S1l: Outcomes of the NMA with comparisons versus PPAR-gamma agonists**

**Figure S1m: Outcomes of the NMA with comparisons versus platelet ADP receptor inhibitors**

**Figure S2: Forest graph showing the relative efficacies of various drug classes in reducing cIMT progression observed in the placebo-controlled studies (no comparator drug) network meta-analysis.**

**
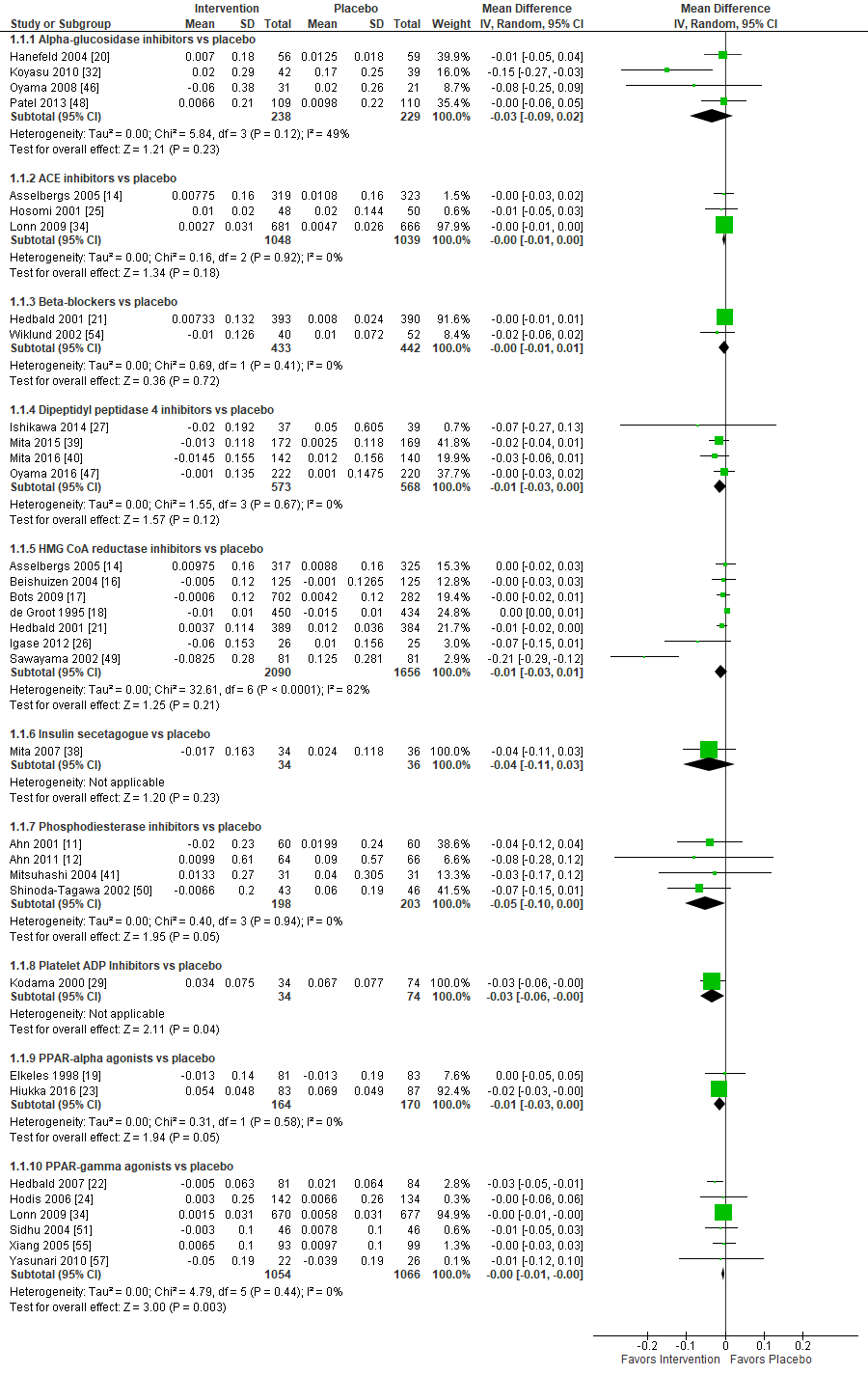
**

**Figure S3: Relative efficacies of several drug classes in reducing cIMT progression as observed in the placebo-controlled studies. Data represent a random effects conventional meta-analysis of mean differences between active drug and placebo.**

**Figure S4: A funnel plot indicating no significant publication bias (Begg’s adjusted Kendall's Score = -157, Std. Dev.= 105.62; p=0.137)**

**Appendix S1 (Literature search Keyword combinations)**

1. carotid intima media thickness – atherosclerosis – drug therapy
2. carotid intima media thickness – atherosclerosis – drug therapy - alpha-glucosidase inhibitors
3. carotid intima media thickness – atherosclerosis – drug therapy - angiotensin inhibitors
4. carotid intima media thickness – atherosclerosis – drug therapy - beta-adrenergic receptor antagonists
5. carotid intima media thickness – atherosclerosis – drug therapy – biguanides
6. carotid intima media thickness – atherosclerosis – drug therapy - cyclooxygenase inhibitors, calcium channel blockers
7. carotid intima media thickness – atherosclerosis – drug therapy - dipeptidyl peptidase-4 inhibitors
8. carotid intima media thickness – atherosclerosis – drug therapy - hydroxy-methylglutaryl coenzyme A reductase inhibitors
9. carotid intima media thickness – atherosclerosis – drug therapy – statin
10. carotid intima media thickness – atherosclerosis – drug therapy - insulin secretagogues
11. carotid intima media thickness – atherosclerosis – drug therapy - peroxisome proliferator-activated receptor agonists
12. carotid intima media thickness – atherosclerosis – drug therapy - phosphodiesterase inhibitors
13. carotid intima media thickness – atherosclerosis – drug therapy - platelet adenosine diphosphate inhibitors
14. carotid intima media thickness – atherosclerosis – drug therapy – diabetes
15. carotid intima media thickness – atherosclerosis – drug therapy – hypertension
16. carotid intima media thickness – atherosclerosis – drug therapy – dyslipidemia
17. carotid intima media thickness – atherosclerosis – drug therapy – hypercholesterolemia
18. carotid intima media thickness – atherosclerosis – drug therapy - anti-diabetic therapy
19. carotid intima media thickness – atherosclerosis – drug therapy - anti-hypertensive therapy
20. carotid intima media thickness – atherosclerosis – drug therapy - randomized trial.
